# Inhibition of the NOD-Like Receptor Protein 3 Inflammasome Is Protective in Juvenile Influenza A Virus Infection

**DOI:** 10.3389/fimmu.2017.00782

**Published:** 2017-07-10

**Authors:** Bria M. Coates, Kelly L. Staricha, Nandini Ravindran, Clarissa M. Koch, Yuan Cheng, Jennifer M. Davis, Dale K. Shumaker, Karen M. Ridge

**Affiliations:** ^1^Department of Pediatrics, Feinberg School of Medicine, Northwestern University, Chicago, IL, United States; ^2^Ann & Robert H. Lurie Children’s Hospital of Chicago, Chicago, IL, United States; ^3^Department of Medicine, Feinberg School of Medicine, Northwestern University, Chicago, IL, United States; ^4^Department of Cell and Molecular Biology, Feinberg School of Medicine, Northwestern University, Chicago, IL, United States

**Keywords:** children, influenza, inflammasome, inflammation, MCC950, acute lung injury

## Abstract

Influenza A virus (IAV) is a significant cause of life-threatening lower respiratory tract infections in children. Antiviral therapy is the mainstay of treatment, but its effectiveness in this age group has been questioned. In addition, damage inflicted on the lungs by the immune response to the virus may be as important to the development of severe lung injury during IAV infection as the cytotoxic effects of the virus itself. A crucial step in the immune response to IAV is activation of the NOD-like receptor protein 3 (NLRP3) inflammasome and the subsequent secretion of the inflammatory cytokines, interleukin-1β (IL-1β), and interleukin-18 (IL-18). The IAV matrix 2 proton channel (M2) has been shown to be an important activator of the NLRP3 inflammasome during IAV infection. We sought to interrupt this ion channel-mediated activation of the NLRP3 inflammasome through inhibition of NLRP3 or the cytokine downstream from its activation, IL-1β. Using our juvenile mouse model of IAV infection, we show that inhibition of the NLRP3 inflammasome with the small molecule inhibitor, MCC950, beginning 3 days after infection with IAV, improves survival in juvenile mice. Treatment with MCC950 reduces NLRP3 levels in lung homogenates, decreases IL-18 secretion into the alveolar space, and inhibits NLRP3 inflammasome activation in alveolar macrophages. Importantly, inhibition of the NLRP3 inflammasome with MCC950 does not impair viral clearance. In contrast, inhibition of IL-1β signaling with the IL-1 receptor antagonist, anakinra, is insufficient to protect juvenile mice from IAV. Our findings suggest that targeting the NLRP3 inflammasome in juvenile IAV infection may improve disease outcomes in this age group.

## Introduction

Influenza A virus (IAV) is a significant respiratory pathogen in the pediatric age group. Despite widespread vaccination efforts, ~80 per 100,000 children in the United States are hospitalized each year with seasonal IAV (1), and up to 24% of hospitalizations require intensive care unit admission for life-threatening disease (2). Underlying medical conditions increase the risk of severe IAV infection, but a considerable amount of morbidity and mortality occurs in healthy children. The effectiveness of antiviral drugs, which target IAV proteins, is hindered by the need to administer them early in the course of infection and the increasing resistance of seasonal IAV to these compounds (3). Consequently, therapy for children with severe IAV infection largely consists of supportive care. Hence, there is an urgent need to develop new therapeutic strategies to reduce the fatal pathology observed in children hospitalized with severe IAV infection.

The host immune response to IAV plays an important role in reducing morbidity and mortality as well as promoting viral clearance. However, IAV infections in pediatric patients can be associated with aberrant or dysregulated cytokine and cellular inflammatory responses [reviewed in Ref. (4)]. Among the potentially injurious cytokines produced during IAV infection are interleukin-1β (IL-1β) and interleukin-18 (IL-18), which are secreted following activation of the NOD-like receptor family pyrin domain containing 3 (NLRP3) inflammasome. The NLRP3 inflammasome is tightly regulated, requiring two signals for activation. Signal 1 occurs through pathogen detection by pattern recognition receptors that act through the transcription factor, NF-κB, to increase the expression of pro-IL-1β, as well as inflammasome components, including NLRP3 and pro-caspase-1. A second signal is then required for NRLP3 inflammasome complex assembly and activation. Several IAV-specific products have been identified as potent Signal 2 activators, including IAV viral RNA and IAV matrix 2 (M2) protein (5–7). The IAV M2 protein, a proton channel involved in viral replication (8), has been shown to activate the NLRP3 inflammasome in an IAV strain-independent manner (7). In bone marrow derived macrophages primed with lipopolysaccharide, which activates Signal 1, lentivirus expression of the M2 protein from a number of seasonal and pandemic strains of IAV resulted in IL-1β secretion. The IAV M2 protein activates the inflammasome by promoting proton efflux following M2 localization to the acidified Golgi apparatus. Inhibition of M2 protein function *via* the introduction of M2 mutants, or the use of amantadine or rimantadine, which block movement of protons through the M2 channel, has also been shown to inhibit IL-1β maturation and secretion (7). Unfortunately, the development of resistance to these medications has diminished their efficacy in the treatment of seasonal IAV, and they are no longer considered standard of care in the United States.

The role of NLRP3 inflammasome in IAV infection was first explored in mice deficient in its three components, NLRP3, caspase-1, or ASC. Decreased survival was consistently seen in mice lacking caspase-1 or ASC when challenged with IAV (9–11). However, the role of the NLRP3 protein itself appeared to be dependent on the inoculating dose of IAV, as NLRP3 deficiency did not impact mortality when a low dose of IAV was used (11), but did lead to worse survival after infection with higher doses (9, 10). The authors reasoned that the increased mortality seen in mice deficient in NLRP3 inflammasome components was due to impaired viral clearance, as viral titers remained elevated late in infection (9, 11). Conversely, more recent studies have demonstrated that excessive NLRP3 inflammasome activity can contribute to IAV-induced lung injury and death (6, 12). Therefore, NLRP3 inflammasome activity must be carefully controlled to achieve IAV clearance without causing unnecessary damage to surrounding tissues. This pathway may be of particular importance in the pathogenesis of severe IAV infection in children (4, 13). Therefore, using our mouse model of pediatric IAV infection, which has been shown to mimic human disease, we investigated how modulation of the inflammatory response might change outcomes in life-threatening IAV infection. Using a small molecule inhibitor of the NLRP3 inflammasome (MCC950) and an antagonist of the receptor for IL-1β (anakinra), we found that inhibition of the NLRP3 inflammasome could ameliorate life-threatening IAV infection in juvenile mice, but inhibition of IL-1β signaling alone could not.

## Materials and Methods

### Animals

129S wild-type mice were provided by Jackson Laboratories and bred in house. Mice were provided with food and water *ad libitum*, maintained on a 14 h light, 10 h dark cycle, and handled according to the National Institutes of Health guidelines. All procedures complied with federal guidelines and were approved by The Institutional Animal Care and Use Committee at Northwestern University.

### Virus

Influenza virus strain A/WSN/1933 (WSN) was grown for 48 h at 37.5°C and 50% humidity in the allantoic cavities of 10- to 11-day-old fertile chicken eggs. Viral titers were measured by plaque assay in Madin–Darby canine kidney (MDCK) epithelial cells. Virus aliquots were stored in liquid nitrogen, and freeze/thaw cycles were avoided.

### *In Vitro* Influenza Virus Infection of THP-1 Cells

THP-1 cells were plated in 6-well plates at a density of 0.5 × 10^6^ per well. They were differentiated with phorbol myristate acetate (5 nM) for 48 h and cultured in complete RPMI medium for 72 h. Cells were infected with IAV WSN at a multiplicity of infection (MOI) of 1, 2, or 3 for 2 h. Cells were then washed with phosphate-buffered saline (PBS) and cultured in complete RPMI medium for 24 h. The cell-free supernatant was collected for ELISA. For drug-therapy experiments, differentiated THP-1 cells were treated with MCC950 (1 µM, Adipogen), anakinra (0.5 µg/mL, Kineret™), or vehicle control (PBS) for 1 h. Immediately after the drug treatment, cells were infected with IAV WSN (MOI 2) for 2 h. The infected cells were washed with PBS and cultured in complete RPMI medium containing MCC950 (1 µM), anakinra (0.5 µg/mL), or vehicle control for 24 h. The cell-free supernatant was collected for ELISA. ELISA was done for IL-1β (eBioscience, San Diego, CA, USA) and Caspase-1 (R&D Systems, Minneapolis, MN, USA).

### Cell Imaging

Human THP-1 monocytes were plated on sterilized 18CIR-1 coverglasses in a 12-well plate at a density of 0.25–0.5 million cells per well and differentiated. Cells were then treated with an MOI of 2 of IAV for 24 h and probed for active caspase-1 by means of FAM-YVAD-FMK (FAM-FLICA caspase-1 assay kit #97, ImmunoChemistry, Bloomington, MN, USA) according to the manufacturer’s instructions. Nuclei were labeled with Hoechst 33342, and then cells were fixed in 2.7% paraformaldehyde for 5 min at room temperature. Images were acquired by means of a Nikon A1R laser scanning confocal microscope.

### *In Vivo* Influenza Virus Infection

Juvenile (4-week-old) mice were anesthetized with isoflurane and infected intratracheally with WSN [12.5 plaque forming units (PFU) in 50 μL PBS] or an equal volume of PBS.

### Inflammasome Inhibition *In Vivo*

MCC950 (Adipogen) reconstituted in sterile PBS was administered intraperitoneally in juvenile mice at a dose of 10 mg/kg daily beginning on day 3 postinfection (p.i.) until tissue harvest, death, or recovery. Anakinra (Kineret™) was administered intraperitoneally in juvenile mice at a dose of 100 mg/kg on day 3 p.i. until tissue harvest, death, or recovery.

### Bronchoalveolar Lavage Fluid (BALF) Harvest

A 20-gauge angiocatheter was ligated into the trachea, and the lungs were lavaged twice with sterile PBS (700 µL). The lavage fluid was centrifuged at 1,000 *g* for 10 min. The pellet was resuspended, and the cells were counted using the Invitrogen Countess Automated Cell Counter (Invitrogen, Grand Island, NY, USA). Protein levels in the supernatant were measured by Bradford Assay (BioRad), and cytokine levels were measured using ELISA. Interleukin (IL)-18 was measured using the mouse IL-18 ELISA Kit (MBL International Corporation, Woburn, MA, USA) according to the manufacturer’s instructions. Interleukin-6 (IL-6) was measured using the mouse IL-6 Ready-Set-GO ELISA Kits (eBioscience, San Diego, CA, USA). Interferon (IFN)-α was measured using the mouse IFN Alpha ELISA Kit (PBL Assay Science, Piscataway, NJ, USA).

### Wet-to-Dry Weight Ratios

Mice were anesthetized and lungs were surgically removed *en bloc*. Lungs were weighed in a tared container. The lungs were then dried at 45°C in a Speed-Vac SC100 evaporator (Thermo Scientific, Waltham, MA, USA) until a constant weight was obtained, and the wet-to-dry weight ratio was calculated.

### Histology

Mice were anesthetized and lungs were perfused *via* the right ventricle with 10 mL HBSS with calcium and magnesium. A 22-gauge angiocatheter was sutured into the trachea, heart and lungs were removed en bloc, and then lungs were inflated with 0.7 mL of 4% paraformaldehyde at a pressure not exceeding 16 cm H_2_O. Tissue was fixed in 4% paraformaldehyde overnight at 4°C, then processed, embedded in paraffin, sectioned, and stained with hematoxylin and eosin (H&E). Images were acquired by means of a TissueGnostics automated slide imaging system (TissueGnostics, Vienna, Austria).

### Lung Harvest and Homogenization

For plaque assay, lungs were homogenized in PBS (20 µL/mg lung). For western blot, lungs were homogenized in RIPA buffer with protease inhibitor (20 mM Tris–HCl, 150 mM NaCl, 1% Triton X-100, 0.1% SDS, Roche complete ULTRA Tablet). Homogenized lungs were centrifuged at 1,000 *g*. The supernatant was frozen at 80°C.

### Western Blot

The presence of indicated proteins in lung homogenates from day 7 p.i. was assessed by western blotting using the following antibodies: NLRP3 (Adipogen), Caspase-1 (14F468) (Santa Cruz sc-56036), ASC (Adipogen), IL-18 (Biovision, 5180R-10), and Actin (Santa Cruz).

### Flow Cytometry for Intracellular Staining of NLRP3 Inflammasome Components

Mice were anesthetized and lungs were perfused *via* the right ventricle with 10 mL HBSS with Ca^2^^+^ and Mg^2^^+^. The lung lobes were removed and inflated with enzyme solution (5 mL of 0.2 mg/mL DNase I and 2 mg/mL Collagenase D in HBSS with Ca^2^^+^ and Mg^2^^+^) using a 30G needle. The tissue was minced and then processed in GentleMACS dissociator (Miltenyi) according to the manufacturer’s instructions. Processed lungs were passed through a 40 µm cell strainer, and red blood cells were lysed with BD Pharm Lyse (BD Biosciences, San Jose, CA, USA). Remaining cells were counted with a Countess Cell Counter (Invitrogen, Grand Island, NY, USA). CD45 microbeads were added, and cells were eluted according to the Miltenyi manufacturer’s instructions. Cells were stained with viability dye Aqua (Invitrogen) and stained with a mixture of fluorochrome-conjugated antibodies (see Table 1 for lists of fluorochromes, antibodies, manufacturers, and clones). Data were acquired on a BD LSR II flow cytometer using BD FACSDiva software (BD Biosciences), and data analyses were performed with FlowJo software (TreeStar, Ashland, OR, USA). Cell populations were identified using sequential gating strategy, and the percentage of cells in the live/singlets gate was multiplied by the number of live cells to obtain an absolute live-cell count. The expression of activation markers is presented as median fluorescence intensity (MFI).

**Table 1:** Fluorochrome-conjugated antibodies used for flow cytometry.

Fluorochrome	Antibody	Manufacturer	Clone
FITC	CD45	eBioscience	30-F11
PerCPCy5.5	MHCII	BioLegend	M5/114.15.2
eFluor450	CD11b	eBioscience	M1/70
Alexa700	Ly6G	BD Pharmingen	1A8
APCCy7	Ly6C	eBioscience	HK1.4
PE	CD64	BioLegend	X54-5/7.1
PECF594	Siglec F	BD Horizon	E50-2440
PECy7	CD11c	BD Pharmingen	HL3

**Additional antibodies used for intracellular staining:**			

**Fluorochrome**	**Antibody**	**Manufacturer**

APC	mNLRP3/NALP3	R&D
Biotin	Caspase-1	NOVUS
APC	Streptavidin	eBioscience

### Plaque Assay

Confluent monolayers of MDCK cells were infected with stock virus or lung homogenate serially diluted in 1% bovine serum albumin Dulbecco’s Modified Eagle Medium (DMEM) for 2 h at 37°C. Plates were washed with PBS and an overlay of 50% 2× Replacement Media (2× DMEM, 0.12 M NaHCO_3_, 2% Penn–Strep, and 1% HEPES), 50% avecil (2.35%), and *N*-acetyl trypsin (1.5 µg/mL) remained on the cells for 72 h at 37°C. Overlay was removed, and the monolayers were then stained with Naphthalene Blue-Black and plaques counted.

### Statistical Analysis

Data are expressed as means ± SD. Differences between two groups were assessed by using a Student’s *t*-test. Differences between three or more groups were assessed using one-way analysis of variance with a Bonferroni multiple comparisons test. Values of *P* < 0.05 were considered to be significant. The log rank test was used in the analysis of the Kaplan–Meier curve. All analyses were performed using GraphPad Prism software version 6.0 for Windows (GraphPad Software, San Diego, CA, USA).

## Results

### MCC950 and Anakinra Decrease NLRP3 Inflammasome Activity *In Vitro* in Macrophages Infected with IAV

MCC950 is a small molecule inhibitor of the NLRP3 inflammasome. Although its exact mechanism of action is unknown, it has been shown to be specific to NLRP3 and to prevent the activation of caspase-1 and the maturation and secretion of IL-1β and IL-18 in response to multiple NLRP3 inflammasome stimuli (14–16). Anakinra is a synthetic version of the naturally occurring IL-1β receptor antagonist. It prevents the downstream signaling of the IL-1β receptor. We tested the ability of MCC950 and anakinra to inhibit the NLRP3 inflammasome *in vitro*.

**Figure 1:**
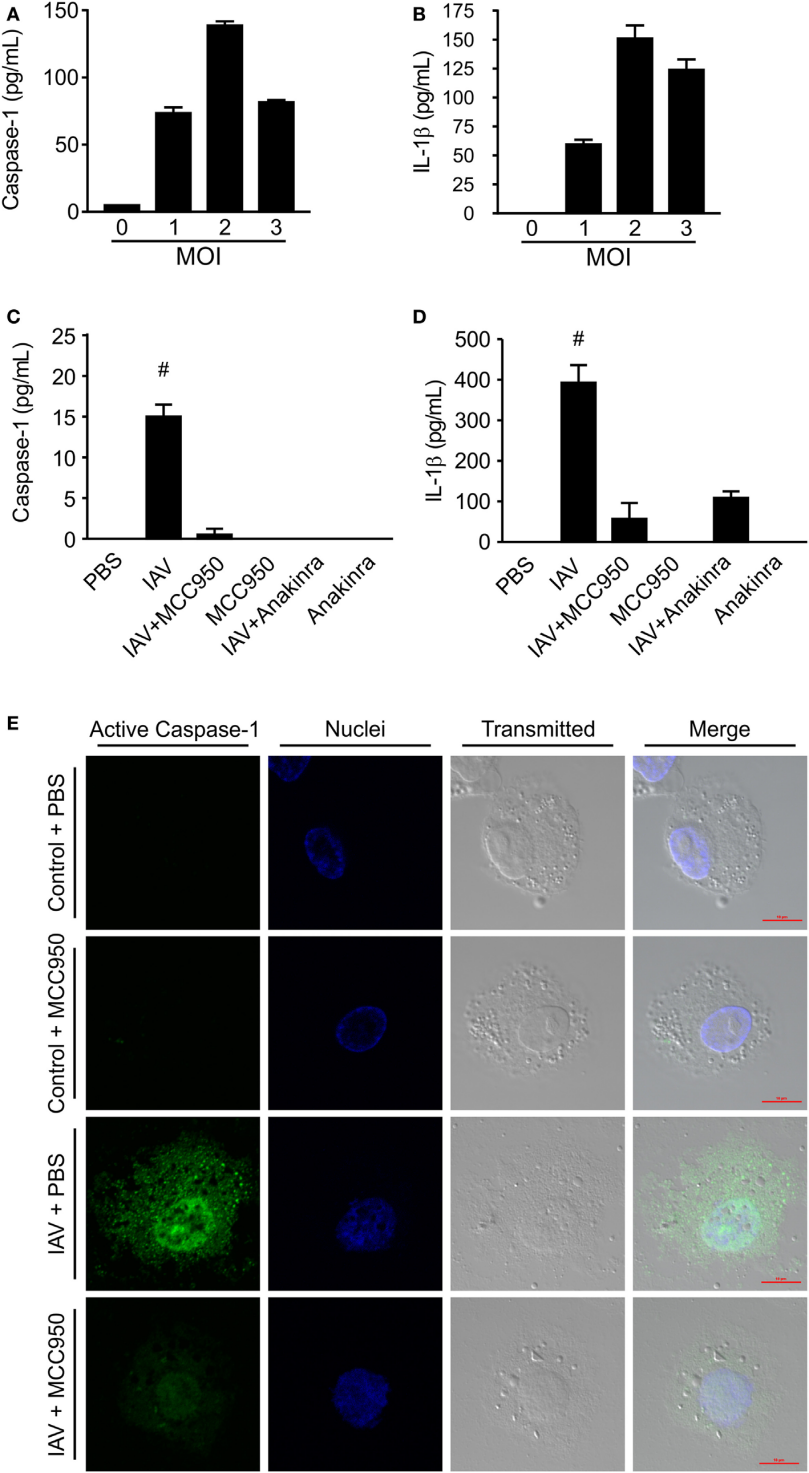
MCC950 and anakinra inhibit influenza A virus (IAV)-induced NOD-like receptor protein 3 inflammasome activation in THP-1 macrophages. Differentiated human THP-1 macrophages were infected with IAV (WSN) at a multiplicity of infection (MOI) of 1, 2, or 3 for 2 h. Infected cells were cultured for 24 h, and supernatant was evaluated by ELISA for **(A)** caspase-1 or **(B)** interleukin-1β (IL-1β). Differentiated human THP-1 macrophages were treated with MCC950, anakinra, or vehicle control. Cells were either infected with IAV (WSN, MOI 2) for 2 h or sham infected and treated with drug therapy alone. Cells were then washed and cultured in media containing MCC950, anakinra, or vehicle control. Supernatant was collected 24 h after IAV infection and evaluated by ELISA for **(C)** caspase-1 or **(D)** IL-1β. ^#^ indicates significant elevation over all other conditions. **(E)** MCC590-treated or vehicle-treated cells were fixed 24 h following IAV infection (WSN MOI 2) and fluorescently labeled to show active caspase-1 (green) and nuclei (blue). Scale bars, 10 µm.

To determine the IAV inoculation dose necessary for NLRP3 inflammasome activation, we infected THP-1 cells (a human monocyte cell line derived from a 1-year-old patient) with IAV at an MOI of 1, 2, and 3 for 24 h. As shown in Figures 1A,B, there was a dose dependent increase in caspase-1 and IL-1β levels in the supernatant from IAV-infected macrophage cells. Based on these results we chose to test the ability of MCC950 and anakinra to inhibit NLRP3 inflammasome activation in response IAV at an MOI of 2.

THP-1 cells were pretreated with MCC950 (1 µM) or anakinra (0.5 µg/mL) and then infected with IAV (A/WSN/2009) at an MOI of 2. As shown in Figures 1C,D, cells infected with IAV had a robust increase in caspase-1 and IL-1β in the supernatant. In contrast, when the cells were treated with the NLRP3 inhibitor, MCC950, or the IL-1β receptor antagonist, anakinra, detection of caspase-1 and IL-1β during IAV infection was greatly reduced. Caspase-1 activation was also assessed using a specific fluorescent probe, FAM-YVAD-FMK (17). Cells infected with IAV showed robust caspase-1 activation following treatment with IAV, with caspase-1 forming aggregates throughout the cytoplasm. However, caspase-1 activation was severely reduced in cells treated with MCC950 prior to IAV infection. No caspase-1 activation was observed in uninfected THP-1 cells (Figure 1E).

### MCC950 Improves Survival of Juvenile Mice Infected with IAV

**Figure 2:**
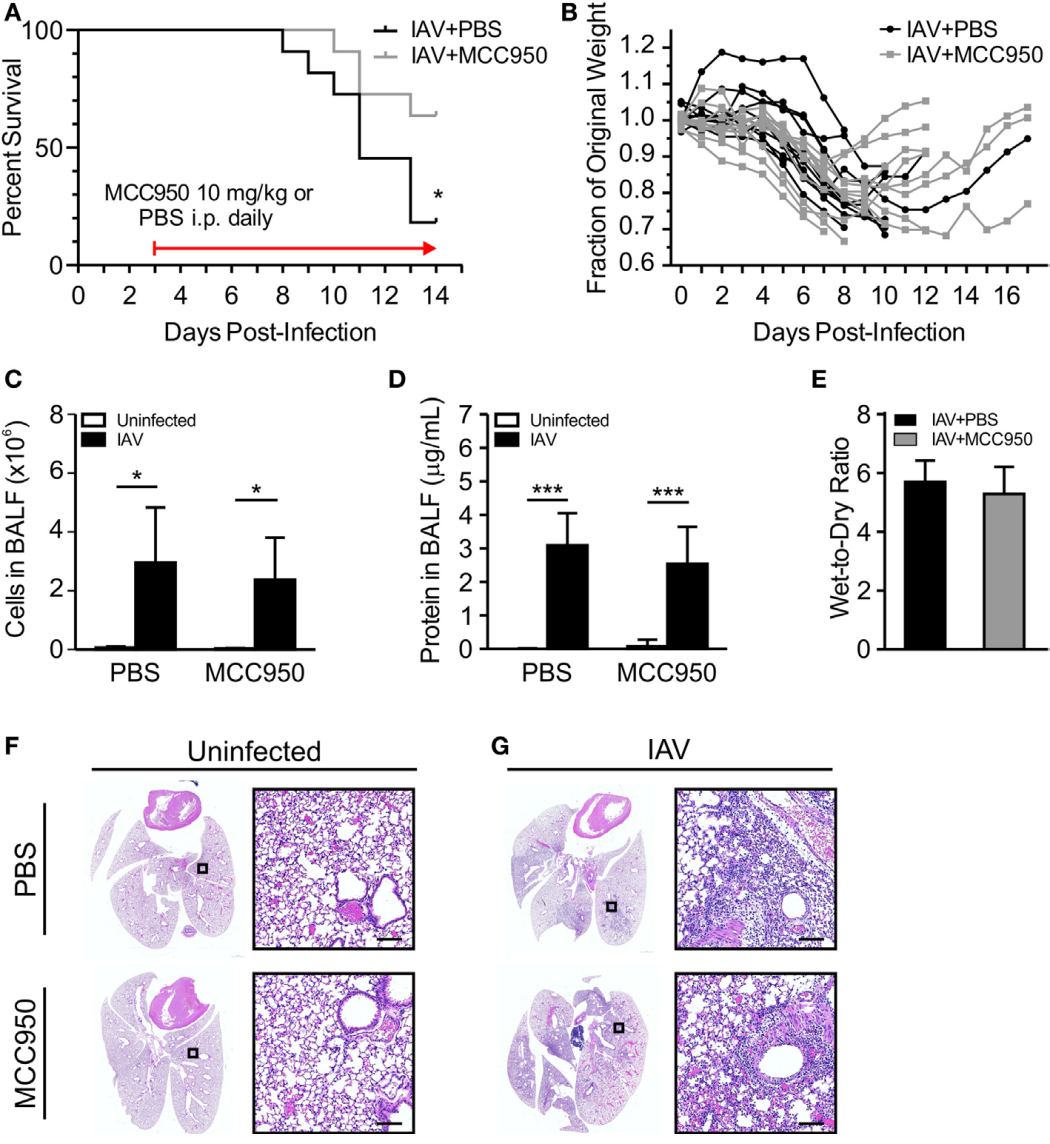
MCC950 improves survival in juvenile mice infected with influenza A virus (IAV). Juvenile mice were infected with IAV [WSN 12.5 plaque forming units (PFU) intratracheal] and treated with MCC950 [10 mg/kg intraperitoneal (i.p.) daily] or phosphate-buffered saline (PBS) control beginning on day 3 postinfection (p.i.). **(A)** Mortality. **(B)** Weight loss. Bronchoalveolar lavage fluid (BALF) or whole lungs were collected from IAV-infected, MCC950-treated mice and IAV-infected, PBS-treated mice 7 days p.i. **(C)** Total number of cells in BALF. **(D)** Protein in BALF. **(E)** Wet-to-dry weight ratio. *p < 0.05, ***p < 0.001. **(F,G)** Hematoxylin and eosin stained lung sections from juvenile mice 7 days p.i. with 12.5 PFU of IAV and treatment with 10 mg/kg MCC950 or PBS control. Images shown are representative of three mice for each condition. Scale bars, 100 µm.

The induction of IL-1β by IAV has been shown to be NLRP3 inflammasome dependent (8–10). We assessed the ability of MCC950 to prevent NLRP3 inflammasome activation and alter the young host’s inflammatory response to IAV infection. Juvenile mice were infected with IAV [A/WSN/2009 12.5 PFU intratracheal (i.t.)] to achieve infection of the lower respiratory tract. Starting on day 3 p.i., we administered MCC950 [10 mg/kg intraperitoneal (i.p.), once daily (q.d.)] or an equal volume of vehicle control (i.p., q.d.) until recovery or death. We observed that the median survival of IAV-infected, vehicle-treated juvenile mice was day 11 p.i, with only 18% of PBS-treated mice surviving infection (Figure 2A). In contrast, 75% of IAV-infected, MCC950-treated juvenile mice were alive on day 11 p.i. IAV infection is typically associated with significant weight loss, which was observed in both IAV-infected, PBS-treated and IAV-infected, MCC950-treated mice. Importantly, the majority of the IAV-infected, MCC950-treated mice began to regain weight between days 8 and 9 p.i. (Figure 2B). Surviving IAV-infected, MCC950-treated juvenile mice exhibited coat ruffling, febrile shaking, and mild lethargy, but the majority of animals recovered. At 7 days p.i., indices of lung injury were not different between IAV-infected, PBS-treated and IAV-infected, MCC950-treated mice. Both groups displayed elevated levels of cellular infiltration (Figure 2C) and protein leakage (Figure 2D) into the BALF, and both groups had increased wet-to-dry weight ratios (Figure 2E). In accordance with this, histological examination of IAV-infected, PBS-treated and IAV-infected, MCC950-treated mice on day 7 p.i. demonstrated a similar degree of lung injury at this time point (Figures 2F,G).

### MCC950 Inhibits the NLRP3 Inflammasome in Juvenile Lungs

**Figure 3:**
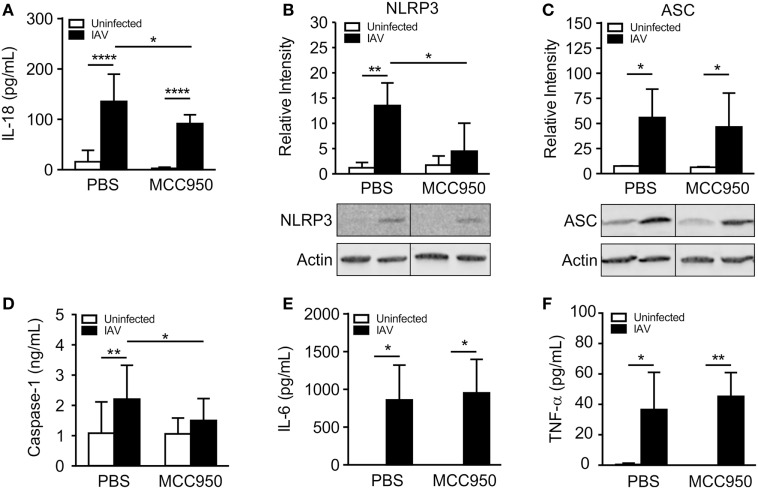
MCC950 treatment decreases NOD-like receptor protein 3 (NLRP3) inflammasome activation in juvenile influenza A virus (IAV) infection. Juvenile mice were infected with IAV (WSN 12.5 plaque forming unit intratracheal) and treated with MCC950 (10 mg/kg intraperitoneal daily) or phosphate-buffered saline (PBS) control beginning on day 3 postinfection (p.i.). Bronchoalveolar lavage fluid (BALF) or whole lungs were collected from IAV-infected, MCC950-treated mice and IAV-infected, PBS-treated mice 7 days p.i. **(A)** Interleukin-18 (IL-18) in BALF as measured by ELISA. **(B,C)** NLRP3 and ASC in lung homogenates as measured by Western blot. **(D)** Caspase-1 in BALF as measured by ELISA. **(E)** Interleukin-6 (IL-6) and **(F)** tumor necrosis-α (TNF-α) in BALF as measured by ELISA. *p < 0.05, **p < 0.01, ****p < 0.0001.

We next sought to compare NLRP3 inflammasome activation in IAV-infected, PBS-treated and IAV-infected, MCC950-treated juvenile mice. IL-18 was elevated in the BALF from IAV-infected, PBS-treated mice, but was significantly attenuated in IAV-infected, MCC950-treated mice (Figure 3A). Importantly, the IAV-induced increase in NLRP3 protein expression observed in PBS-treated mice was absent in MCC950-treated mice (Figure 3B). Additional western blot analysis of inflammasome components in lung homogenates showed a similar increase in ASC in response to IAV infection in both PBS-treated and MCC950-treated mice (Figure 3C). Mature caspase-1 was increased in the BALF from IAV-infected, PBS-treated mice, compared to uninfected controls (Figure 3D). In contrast, caspase-1 secretion was inhibited in IAV-infected, MCC950-treated mice (Figure 3D). IL-6 and tumor necrosis-α (TNF-α), which are inflammatory cytokines that are not dependent on NLRP3 inflammasome activation, were not different between the two treatment groups (Figures 3E,F).

### MCC950 Does Not Prevent Monocyte Recruitment to the Lungs but Does Inhibit NLRP3 Inflammasome Activation in Alveolar Macrophages

**Figure 4:**
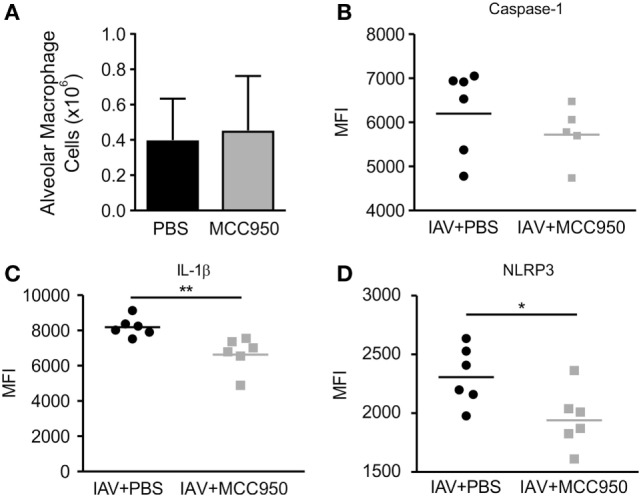
MCC950 treatment decreases NOD-like receptor protein 3 (NLRP3) inflammasome activation in alveolar macrophages in juvenile mice infected with influenza A virus (IAV). Juvenile mice were infected with IAV (WSN 12.5 plaque forming unit intratracheal) and treated with MCC950 (10 mg/kg intraperitoneal daily) or phosphate-buffered saline (PBS) control beginning on day 3 postinfection (p.i.). Lungs were harvested 7 days p.i. and evaluated by flow cytometry for NLRP3 inflammasome activation using intracellular staining. **(A)** Number of alveolar macrophages in lung homogenates. **(B–D)** Median fluorescence intensity (MFI) of caspase-1, interleukin-1β (IL-1β), and NLRP3 in alveolar macrophages. *p < 0.05, **p < 0.01.

Macrophages are a main source of NLRP3 inflammasome activation during IAV infection (18). To investigate the impact of MCC950 treatment on NLRP3 inflammasome activation in these cells, we isolated alveolar macrophages (CD45+, CD64+, CD11c+, Siglec F+) and monocyte-derived cells (CD45+, CD11b+, Ly6C+, CD64+) from the lungs of IAV-infected, PBS-treated and IAV-infected, MCC950-treated mice using 10-color flow cytometry (19). In addition, we assessed the expression levels of NLRP3, caspase-1, and IL-1β with intracellular staining. IAV infection caused an influx of monocyte-derived cells into the lungs. When analyzing all CD45+ cells, a similar number of alveolar macrophages (Figure 4A) and monocyte-derived cells (data not shown) were found in both treatment groups on day 7 p.i. To determine if MCC950 inhibited the expression of NLRP3 in these cells, we examined the median fluorescence intensity (MFI) of the inflammasome components, NLRP3 and caspase-1, and the product of its activation, IL-1β. All components of the NLRP3 inflammasome measured were significantly elevated in the alveolar macrophages of IAV-infected mice compared to uninfected controls (data not shown and Figures 4B–D). IAV-infected, MCC950-treated mice had significantly decreased levels of NLRP3 and IL-1β in alveolar macrophages (Figures 4C,D). These results are consistent with our finding that IAV-infected, MCC950-treated mice had decreased levels of NLRP3 in homogenized lungs as measured by Western blot, and IL-18 in BALF as measured by ELISA (see Figure 3).

### MCC950 Does Not Impact Type I Interferon Production or Viral Clearance in Juvenile IAV Infection

**Figure 5:**
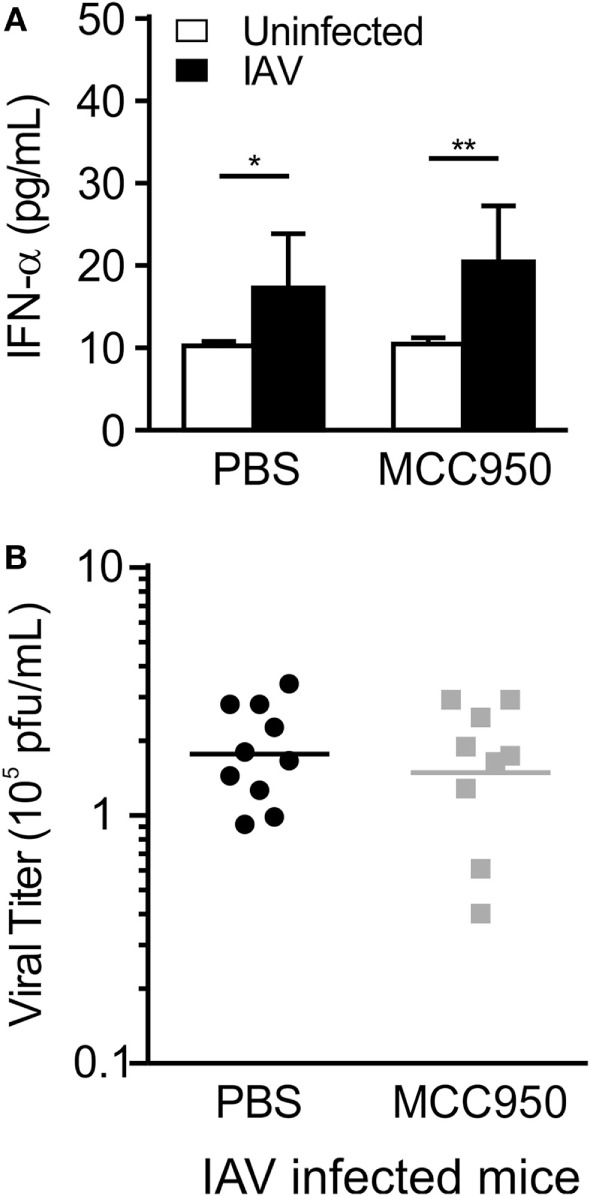
MCC950 treatment does not impair viral clearance. Juvenile mice were infected with influenza A virus (IAV) (WSN 12.5 plaque forming unit intratracheal) and treated with MCC950 (10 mg/kg intraperitoneal daily) or phosphate-buffered saline (PBS) control beginning on day 3 postinfection (p.i.). Bronchoalveolar lavage fluid (BALF) or whole lungs were collected from IAV-infected, MCC950-treated mice and IAV-infected, PBS-treated mice 7 days p.i. **(A)** Interferon-α (IFN-α) in BALF as measured by ELISA. **(B)** Viral titer in lung homogenates was measured by plaque assay. *p < 0.05, **p < 0.01.

Interferon-α (IFN-α) is a type I interferon secreted in response to viral infection to control viral replication and prevent propagation of the infection to neighboring cells. Mice infected with IAV, as well as mice treated with MCC950, had an increase in IFN-α levels on day 7 p.i. (Figure 5A). Consistent with this, viral titers in the lung homogenates of IAV-infected, PBS-treated and IAV-infected, MCC950-treated mice as measured by plaque assay were equal (Figure 5B).

### Anakinra Does Not Protect Juvenile Mice from IAV Infection

**Figure 6:**
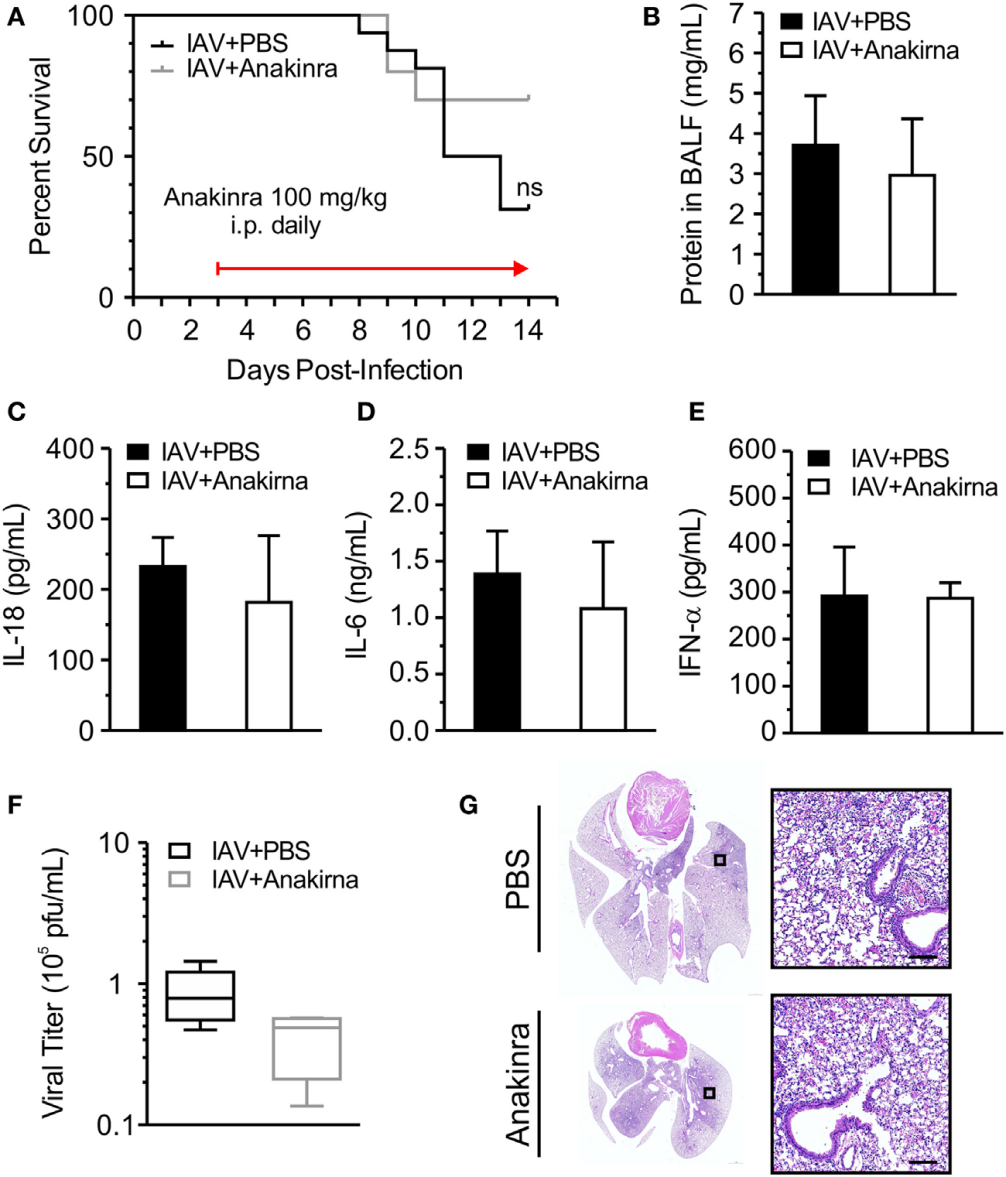
Anakinra treatment does not protect juvenile mice from influenza A virus (IAV) infection. Juvenile mice were infected with IAV (WSN 12.5 plaque forming unit intratracheal) and treated with anakinra [100 mg/kg intraperitoneal (i.p.) daily] or vehicle control beginning on day 3 postinfection (p.i.). **(A)** Mortality. **(B)** Protein in bronchoalveolar lavage fluid (BALF) on day 7 p.i. **(C–E)** Interleukin-18 (IL-18), interleukin-6 (IL-6), and interferon-α (IFN-α) in BALF on day 7 p.i. as measured by ELISA. **(F)** Viral titer on day 7 p.i. was measured by plaque assay. **(G)** Hematoxylin and eosin stained lung sections from juvenile mice 7 days p.i. with IAV and treatment with anakinra or phosphate-buffered saline (PBS) control. Images shown are representative of three mice for each condition. Scale bars, 100 µm.

NOD-like receptor protein 3-dependent production of IL-1β and IL-18 may have downstream consequences with regard to IAV-induced inflammation and disease. IL-1β and IL-18 bind their cell-surface receptors (IL-1R and IL-18R, respectively) expressed on a range of cell types to induce potent NF-κB-dependent secondary cytokine production (20, 21). Importantly, lack of IL-1R resulted in reduced lung immunopathology following H1N1 infection, suggesting that IL-1R signaling may increase damage to the lung (22). Anakinra competes for the IL-1 receptor and blocks the actions of IL-1β. We investigated the impact of anakinra treatment on survival in IAV-infected juvenile mice. Juvenile mice were infected with IAV (A/WSN/2009 12.5 PFU i.t.) and anakinra (100 mg/kg i.p.) or an equal volume of vehicle control was administered i.p., q.d. beginning on day 3 p.i. and continuing until recovery or death. There was no statistically significant difference in the survival of IAV-infected mice treated with anakinra compared to those given vehicle control (Figure 6A). Initiating anakinra therapy on day 2 p.i. or day 4 p.i. also did not improve survival (data not shown). Measurement of protein leakage, IL-18, and IL-6 in BALF failed to show a difference between anakinra and control-treated mice on day 7 p.i. (Figures 6B–D). IFN-α secretion was not impacted by anakinra therapy, either, and viral titers from lung homogenates were equal in IAV-infected anakinra-treated mice, and IAV-infected PBS control-treated mice (Figures 6E,F). Finally, a similar degree of lung injury was seen on histological examination of IAV-infected, PBS-treated and IAV-infected, anakinra-treated mice on day 7 p.i. (Figure 6G).

## Discussion

The host response to IAV can exacerbate the morbidity associated with IAV infection (4, 23, 24). It is well established that the NLRP3 inflammasome is a major component of the host response to IAV (6). It is activated by the influenza M2 proton channel and results in the production of the potent inflammatory cytokines, IL-1β and IL-18 (7). While pathogen clearance and host survival depend on adequate activation of the innate immune system, an excessive inflammatory response to infection can be harmful to the young host. The NLRP3 inflammasome is protective in lethal mouse models of IAV infection (9–11). Loss of NLRP3, ASC, or caspase-1 in mice leads to decreased IL-1β and IL-18 secretion and increases mortality from IAV. Alternatively, excessive inflammasome activation may decrease survival by exacerbating the lung injury seen in lethal IAV infection (6). Therefore, inflammasome signaling must be tightly controlled to promote eradication of the virus while limiting collateral damage to the host. In severe IAV infection, this balance is not achieved, making the NLRP3 inflammasome an attractive therapeutic target. Early modulation of its activity may not only limit the production of injurious inflammatory cytokines, but also prevent the pyroptotic cell death and the ensuing tissue destruction caused by activated caspase-1. Thus, the identification of small molecule inhibitors of the NLRP3 inflammasome offers considerable therapeutic promise. To establish the optimal degree of NLRP3 inflammasome activation during IAV infection, we sought to modulate NLRP3 inflammasome signaling, with the goal of protecting juvenile mice from IAV-induced lung injury. We investigated the efficacy of MCC950, a potent inhibitor of NLRP3, as well as anakinra, a known inhibitor of the IL-1β pathway *via* IL-1 receptor.

MCC950 has been shown to be a specific NLRP3 inhibitor and to be protective in multiple models of injurious NLRP3 inflammasome activation (14–16). It can be given by oral, intravenous, and i.p. routes and is effective at doses ranging from 4 to 20 mg/kg in mouse models of autoimmune disease (experimental autoimmune encephalitis) (14), diseases of constitutive NLRP3 activation (cryopyrin-associated periodic syndrome) (14), and disorders in which NLRP3 has been shown to play an important role [including cardiac infarction (25) and non-alcoholic steatohepatitis (26)]. *In vitro* treatment of IAV-infected THP-1 macrophages with MCC950 confirmed that IAV-induced NLRP3 inflammasome activation is effectively inhibited with this molecule. Interestingly, treatment of IAV-infected cells with the IL-1β receptor antagonist, anakinra, did not just block downstream signaling from the IL-1β receptor. It also appeared to inhibit NLRP3 inflammasome activation, as demonstrated by decreased caspase-1 and IL-1β in the supernatant from IAV-infected, anakinra-treated cells. Although anakinra classically targets IL-1β signaling by blocking the interaction of IL-1β with its receptor, it has also been shown to bind to and inhibit caspase-1, which likely explains our finding of NLRP3 inflammasome inhibition in IAV-infected cells treated with anakinra (27, 28). Consequently, we hypothesized that both MCC950 and anakinra had the potential to protect juvenile mice from IAV-induced NLRP3 inflammasome activation and lung injury.

Juvenile mice treated with MCC950 beginning 3 days p.i. were protected from IAV-induced mortality. We chose this timing for the initiation of therapy because this likely corresponds to when children infected with IAV develop symptoms and seek medical attention. The protection from IAV-induced mortality was associated with a decreased amount of NLRP3 in the lung homogenates, and decreased IL-18 levels in the BALF, from IAV-infected, MCC950-treated mice compared to IAV-infected, vehicle-treated mice, indicating that inhibition of the NLRP3 inflammasome was achieved. However, this protection from IAV-induced mortality was not associated with a decrease in traditional markers of lung injury, including cellular infiltration and protein leakage into the alveolar space. Since MCC950 therapy did not prevent IAV-induced lung injury on day 7 p.i., this suggests that MCC950 treatment improved survival by either halting disease progression or enhancing recovery. When CD45+ cells from IAV-infected mice were examined with intracellular staining, the greatest impact of NLRP3 inflammasome inhibition was found in alveolar macrophages. In these cells, MCC950 treatment decreased NLRP3 and IL-1β levels. As alveolar macrophages can promote alveolar epithelial cell repair (24), the switch from an inflammatory to an anti-inflammatory phenotype in alveolar macrophages may play an important role in recovery from IAV. We reason that inhibiting the NLRP3 inflammasome in this cell population may have played a key role in the beneficial effects of MCC950 therapy.

Other groups have shown varying degrees of protection from IAV (12) or the IAV virulence factor PB1-F2 (29) using MCC950 in murine models of adult IAV. Tate et al. were able to delay death from two different strains of IAV (A/PR/8/34 and HKx31) by a few days with MCC950 (5 mg/kg intranasal) treatment. Notably, timing of initiation of MCC950 therapy was important in their model, with early administration of the drug on day 1 p.i. harmful, and late administration after day 3 beneficial. This could be consistent with our proposal that NLRP3 inflammasome inhibition is important for recovery from IAV infection, rather than prevention of IAV-induced lung injury. However, evaluation of lung injury was not performed in their study. They were able to demonstrate prevention of immune cell infiltration into the lungs and decreased cytokine production, including IL-1β, IL-18, TNF-α, and IL-6, in BALF and serum. In our model of juvenile IAV infection, we did not see the same inhibition of immune cell recruitment to the lungs with MCC950 therapy (10 mg/kg i.p.). We also did not see the same suppression of IL-6 or TNF-α production. However, we were not surprised by this finding because these cytokines are not dependent on NLRP3 inflammasome activation. Importantly, we did see evidence of NLRP3 inflammasome inhibition in the resident alveolar macrophages, and decreased IL-18 in BALF, which may have contributed to the increased survival we found in mice treated with MCC950. Differences in our models may explain the disparate findings regarding cellular recruitment and cytokine suppression, highlighting the importance of the microenvironment when modulating the immune response to a pathogen. We administered the drug i.p. instead of intranasal to avoid repeated exposure to anesthesia, but these two delivery methods could result in different concentrations of the drug in the alveolar space. In addition, the half-life of the drug may be altered by delivery method, potentially limiting its efficacy. Perhaps more importantly, we used juvenile mice, which may have a propensity for worse disease (4, 30, 31). Innate immune signaling has been shown to be vary widely depending on age, making age-relevant models critical when studying inflammatory diseases (32–38).

Genetic deletion of NLRP3 inflammasome components leads to worse outcomes in IAV infection (9–11). This argues that abolishment of NLRP3 inflammasome signaling during IAV infection is harmful and that early NLRP3 inflammasome activation is necessary for controlling the infection and viral clearance. The finding from Tate et al. that early inhibition of NLRP3 with MCC950 increases mortality from IAV infection is consistent with this (12). In contrast, late inhibition was protective, which supports our finding that MCC950 treatment beginning 3 days p.i. improved survival in juvenile mice infected with IAV. The sensitivity of the outcome of NLRP3 modulation to timing and degree of inhibition is not unique to IAV infection, but a common theme in inflammatory responses to pathogens, where inadequate inflammation impairs pathogen clearance, but excessive inflammation causes collateral tissue damage and enhanced injury. This emphasizes the need for careful characterization of optimal treatment strategies in clinically relevant, age appropriate, models.

In contrast to our results from MCC950 treatment, anakinra treatment of IAV-infected juvenile mice did not show protection from IAV-induced mortality or lung injury. Despite achieving NLRP3 inflammasome suppression *in vitro*, anakinra therapy did not effectively decrease NLRP3 inflammasome activation or IL-18 secretion into the alveolar space in our *in vivo* model of juvenile IAV infection. Therefore, it was not surprising that we could not demonstrate protection from IAV with anakinra treatment. Instead, it suggests that once daily dosing with i.p. delivery was not sufficient to achieve caspase-1 inhibition. Alternatively, it argues that isolated IL-1 receptor antagonism is insufficient to protect juvenile mice from IAV infection because it leaves IL-18 signaling intact. There is one report of anakinra therapy (100 μg/mouse, intravenous, daily from day 2 to 6 p.i.) improving survival in IAV infection in adult mice (A/PR/8/34) (39), but only mortality was evaluated. Differences in our model, especially the age of the mice, may explain the discrepancy in our findings.

Influenza A virus is a source of significant morbidity and mortality in children, but current therapies are limited to early antiviral treatment and supportive care. There is considerable need for new strategies to improve outcomes in pediatric IAV infection, and the use of juvenile models to test these strategies is critical. Targeting the NLRP3 inflammasome may be beneficial in juvenile IAV infection and does not appear to impact viral clearance. Better understanding of how NLRP3 inflammasome inhibition improves mortality in juvenile IAV infection, and identification of the optimal timing and method of NLRP3 inflammasome inhibition in juvenile IAV infection, deserve further study.

## Ethics Statement

This study was carried out in accordance with United States federal guidelines and was approved by The Institutional Animal Care and Use Committee at Northwestern University.

## Author Contributions

BC contributed to all aspects of this manuscript including experimental design, conduction of experiments, interpretation of data, and manuscript preparation. NR, YC, JD, and DS contributed to the conduction of the experiments, interpretation of data, and manuscript preparation. KS and CK contributed to experimental design, conduction of experiments, interpretation of data, and manuscript preparation. KR contributed to experimental design, interpretation of data, and manuscript preparation.

## Conflict of Interest Statement

The authors declare that the research was conducted in the absence of any commercial or financial relationships that could be construed as a potential conflict of interest.
